# A new statistical approach for China road freight transport at a subnational level

**DOI:** 10.1371/journal.pone.0287983

**Published:** 2023-10-27

**Authors:** Fuyou Huang, Jianbo Wang, Wenying Xie, Bin Chen, Chao Ma

**Affiliations:** 1 Institute of Transportation Development Strategy & Planning of Sichuan Province, Chengdu, China; 2 Road Traffic Safety Laboratory, Sichuan Vocational and Technical College of Communications, Chengdu, China; 3 School of Transportation and Logistics, Southwest Jiaotong University, Chengdu, China; 4 Hubei Key Laboratory of Power System Design and Test for Electrical Vehicle, Hubei University of Arts and Science, Xiangyang, China; 5 School of Automobile and Traffic Engineering, Hubei University of Arts and Sciences, Xiangyang, China; Libyan Academy, LIBYA

## Abstract

Currently, there is a large deviation between official road freight data and real road freight performance at a subnational level in China. In order to deal with this deviation, the new concept of local freight tonnage and ton-kilometers is presented in this paper based on the territoriality principle, where either the origin or the destination of goods transported is local. Also, the statistic procedures and estimation models of the local freight tonnage and ton-kilometers are proposed based on five accessible basic datasets. Finally, an empirical study in Sichuan province of China is conducted. The statistical results show that there is a large amount of local freight transported by local non-commercial trucks and non-local trucks, which is ignored in the existing road freight statistics. Especially, the higher the level of local economic development, the greater the deviation between the official road freight data and the real road freight performance at a subnational level.

## 1 Introduction

Transportation is usually referred to as the lifeblood of economic development. In particular, efficient freight transport activity is a strong driving force for economic growth [1]. The growth of economy is usually accompanied by an increase in terms of freight tonnage and ton-kilometers. As the largest developing country in the world, the China freight transportation system moved more than 52.98 billion tons of goods in 2021 [2]. Note that approximately three-fourths of goods are shipped by trucks, and road freight transportation remains dominant across all freight transportation modes. The similar situation exists in the United States, the European Union and other countries [3–5]. For example, Trucks carried 60.8 percent of the weight of all goods shipped in the United States in 2018 [6].

Freight data, such as tonnage and ton-kilometers, are not only the basis for measuring the performance of freight transportation development, but also the main reference for the formulation of various transportation plans, policies and infrastructure investments [7]. What’s more, freight tonnage and ton-kilometers are important indexes reflecting the development level of national economy. Governments all over the world attach great importance to freight statistics. For example, in order to assure that statistics provide effective support for transportation decision making in the United States, the Bureau of Transportation Statistics has been publishing the Transportation Statistics Annual Report each year since 1994 based on the commodity flow survey conducted every five years as a part of the Economic Census. The value, tonnage, ton-miles, origin, destination and type of goods can be got via the commodity flow survey. In Korea, the commodity flow survey is also conducted every five years to investigate commodity flow characteristics and to compile statistics for goods movement since 1998 [8].

In China, the Ministry of Transport organized some authoritative institutions to carry out statistical research, and enacted the earliest statistical approach and scheme for highways and waterways in 1993. Since then, the statistical scheme has undergone many improvements and adjustments. Nowadays, the important freight data, such as tonnage and ton-kilometers, are published jointly by the National Bureau of Statistics and the Ministry of Transport. It is worth noting that the published official road freight data is obtained based on the registration place of trucks, which is feasible and effective at the national level. However, the registration place and the operating place of trucks may be different, which leads to serious deviation in the official freight statistics at a subnational level such as a province. For example, the freight tonnage published by Sichuan Statistical Yearbook, includes that shipped by the local trucks (registered in Sichuan province) running anywhere in Sichuan province as well as outside. Even though neither the loading place nor the unloading place of trucks is in Sichuan province, the corresponding tonnage of goods transported is included in the official road freight data of Sichuan province. Meanwhile, it is also worth noting that the published official road freight tonnage only includes that transported by commercial trucks. In Sichuan province, there are a large number of non-commercial trucks and non-local trucks (registered in other provinces) engaging in freight transport activities. The tonnage of goods transported by those trucks is closely related to the economic activities of Sichuan province, but is ignored in the current freight statistics. In summary, the current statistical method is difficult to truly mirror the operations of the subnational road freight system and to show the contributions of road freight to local economy, further weakening the objectivity and rationality of subsequent planning and policy making.

In fact, Xie and Zhang [9] pointed out the shortcomings of the existing statistical method at a subnational level in 2009, they confirmed that it is very necessary to carry out statistics on freight transport based on the territoriality principle, where either the loading place or the unloading place of trucks is local. But they did not establish a feasible statistical method. So far, although there are a large number of studies on road freight statistics for getting more accurate and objective freight information, most of them mainly focus on the improvement of scheme and the optimization of key parameters based on the existing statistical method. For instance, Liu et al. [10] reestablished a calculation model of monthly fluctuation coefficient on road freight tonnage using data from multi-service transport platforms. Liang and Wu [11] claimed that the method of stratified sampling based on vehicles is difficult to last for a long time in the current official statistical scheme, and it is not representative to calculate the freight tonnage of an entire regional road network only by the expressways toll data. Then, they put forward an improved scheme by establishing a grey correlation analysis model.

In recent years, a handful of studies have begun to explore some new ways of road freight statistics. Duan et al. [12] proposed an improved statistical method of freight transport in regional road network based on the full sample data of expressway toll system and the highway traffic survey data, where the average daily truck flow in cross-section and the equivalent mileage are used as two analogical indexes. Zhang et al. [13] developed two automated statistical methods for road freight based on the data fusion principle of fixed and floating detection, where the journey of trucks can be identified by the navigation and positioning system like BeiDou and GPS. And they took Liaoning province as examples to provide statistical results. The improved statistical methods of the above two studies are workable, but cannot mirror the whole picture of trucks in the road network because which rely either on the expressway toll data or on the freight characteristics of floating trucks. Yan et al. [14] proposed the concept of region-oriented freight tonnage, and showed that the proposed statistical method is feasible by comparing the correlation between the region-oriented freight performance and the local GDP. Nevertheless, their statistical methods cannot be implemented at will, and the statistical results are not timely because which are based on the national special survey data of road freight conducted every five years. In addition, there are some optimized methods and innovations on road freight statistics among nations ([15–19]), but the optimized statistical methods cannot be used directly or suitable in China because of different statistical systems and rules.

Motivated by the great deviation between the official road freight data and the real road freight performance at a subnational level, the purpose of this paper is to develop a simple and feasible approach for road freight statistics based on the territoriality principle, where either the loading place or the unloading place of trucks is in a specific region such as a province. In doing so, the improved freight data obtained by the new statistical approach can more accurately and objectively reflect the regional freight transportation situation at a subnational level, and which are closely related to local economic activities. In particular, in order to ensure that the proposed statistical approach is easy to implement, the basic multi-source datasets used in the estimation models are also easy to obtain. Finally, an empirical study with Sichuan province as an example is conducted, and explore the difference between the official freight data and the improved freight data obtained by the new statistical approach.

The remainder of this paper is organized as follows. The next section proposes the new concept of the local freight tonnage and ton-kilometers, and introduces multi-source datasets used in this paper and corresponding statistic procedures. The estimation models of the local freight tonnage and ton-kilometers are established in Section 3. In Section 4, Sichuan province is used as an example to provide statistical results on the local freight tonnage and ton-kilometers. Conclusions and possible future research are drawn in the last section.

## 2 Statistic framework

**Fig 1:**
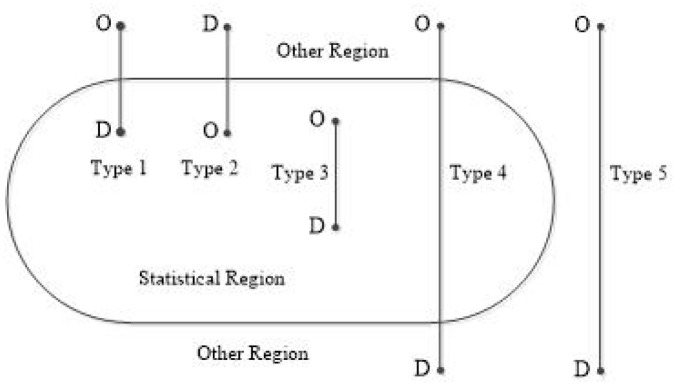
Five types of traffic flows.

In order to objectively reflect the regional road freight transportation situation at a subnational level, a new concept of the local freight tonnage and ton-kilometers based on the territoriality principle is proposed, which is the tonnage and ton-kilometers of goods transported by all the trucks where either the loading place or the unloading place is within a region. Therefore, all the local freight tonnage and ton-kilometers can be closely bonded with the local economy. Fig 1 shows five types of traffic flows, except for the fourth and fifth types, either the origins or the destinations of the other three are in the statistical region. The tonnage and ton-kilometers of goods moved by the three types are local freight tonnage and ton-kilometers based on the territoriality principle. The following sections aim to show how to obtain the local freight tonnage and ton-kilometers.

### 2.1 Basic data

In this subsection, a few basic datasets from industry management departments is introduced, which are crucial for carrying out statistics on the local freight tonnage and ton-kilometers, and are used in our estimation models developed in this paper.

1. Data from the expressway toll system. Almost all expressways are toll roads in China. Once trucks enter any expressway, the tollbooths, times, number of truck axles, license plate number, total weight of vehicle and goods, and distance traveled are recorded automatically in the expressway toll system. All trucks on expressways are divided into five categories by number of truck axles: 2-axle trucks, 3-axle trucks, 4-axle trucks, 5-axle trucks and 6-axle trucks.
2. Data from the traffic monitoring stations. The traffic monitoring stations are widely deployed on the road network, which automatically record the count and types of passing trucks. According to the truck type, all trucks on ordinary roads are divided into four categories: small trucks, medium trucks, heavy trucks and very heavy trucks. Moreover, each traffic monitoring station has the corresponding observation road and observation mileage.
3. Data from the manual sampling survey. Trucks traveling on expressways and on ordinary roads are sampled. It records that the number of truck axles, empty weight, commodities carried, license plate number and origin-destination of the sampled trucks running on expressways, and record the truck type, loads, commodities carried, license plate number and origin-destination of the sampled trucks running on ordinary roads.
4. Data from the local transportation department. The amount, license plate number and other information of all the local commercial trucks can be obtained from the local transportation department. In addition, it can be obtained that the mileage of ordinary roads with different administrative grades in a region.
5. Data from the local statistics yearbook. The tonnage and ton-kilometers of goods transported by all the local commercial trucks can be attained, no matter whether the loading and unloading places for the local commercial trucks are in the statistical area. Meanwhile, the count of the local commercial trucks and the total count of the local trucks from the local statistics yearbook can be obtained.

### 2.2 Statistic procedure

#### 2.2.1 Statistic of local freight tonnage

**Fig 2:**
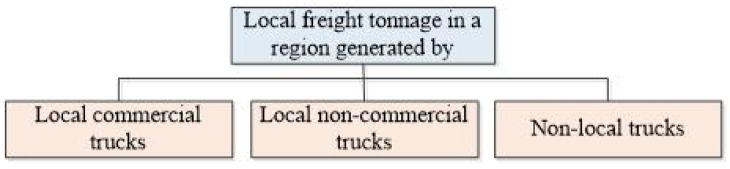
Composition of local freight tonnage.

To avoid double calculation of freight tonnage when a single trip goes over both expressways and ordinary roads, there are no separate estimates of freight tonnage based on highways and general roads. On this account, as shown in Fig 2, the trucks that generate the local freight tonnage are divided into three categories: the local commercial trucks, the local non-commercial trucks and the non-local trucks. Hence, statistics of the local freight tonnage are conducted by these three types of trucks separately, and then sum up to get all the local freight tonnage in a region.

**Fig 3:**
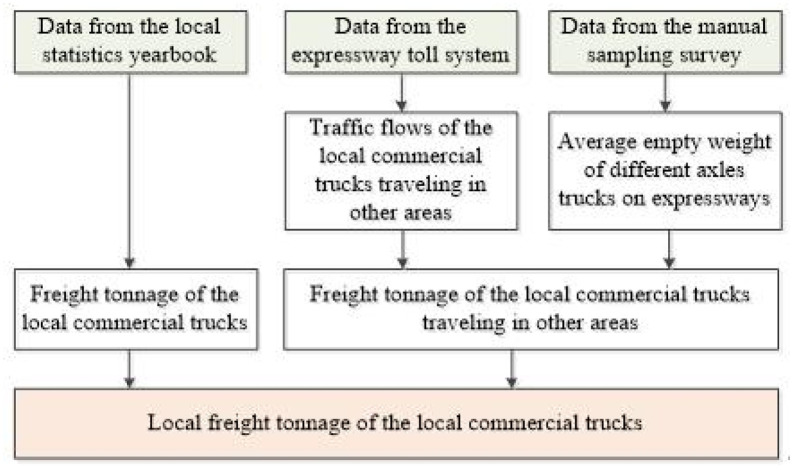
Statistic procedure of the local freight tonnage of local commercial trucks.

Statistics of the local freight tonnage of the local commercial trucks as shown in Fig 3:

Step 1: Identifying the traffic flows of the local commercial trucks running outside the region from the expressway toll system, and extracting key information such as the total weight of vehicle and goods and the number of truck axles.
Step 2: Removing the empty trucks according to the average empty weight of trucks with different number of axles from the manual sampling survey.
Step 3: Estimating the freight tonnage of the local commercial trucks running outside the region.
Step 4: Extracting all the freight tonnage of the local commercial trucks from the local statistics yearbook.
Step 5: Obtaining the local freight tonnage of the local commercial trucks by simple subtraction.

**Fig 4:**
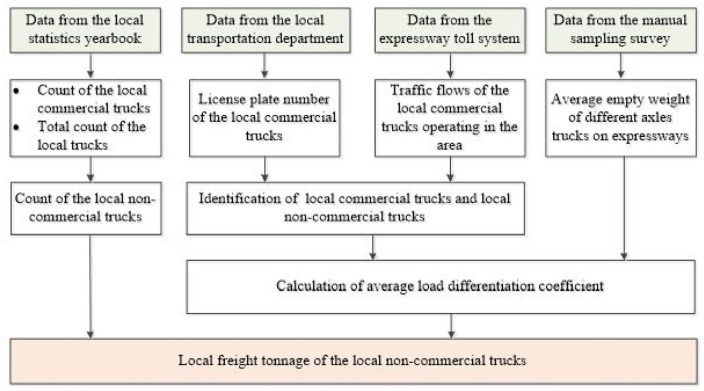
Statistic procedure of the local freight tonnage of local non-commercial trucks.

Statistics of the local freight tonnage of the local non-commercial trucks as shown in Fig 4:

Step 1: Extracting the license plate number of the local commercial trucks from the local transportation department.
Step 2: Extracting the traffic flows of the local commercial trucks and the local non-commercial trucks from the expressway toll system via comparing the license plate number, respectively.
Step 3: Extracting the average empty weight of trucks with different number of axles on expressways from the manual sampling survey.
Step 4: Calculating the average load differentiation coefficient between the local commercial trucks and the local non-commercial trucks based on the data obtained above.
Step 5: Extracting the count of the local commercial trucks and the total count of the local trucks from the local statistics yearbook.
Step 6: Estimating the local freight tonnage of the local non-commercial trucks according to the proportion of local non-commercial trucks and the load differentiation coefficient.

**Fig 5:**
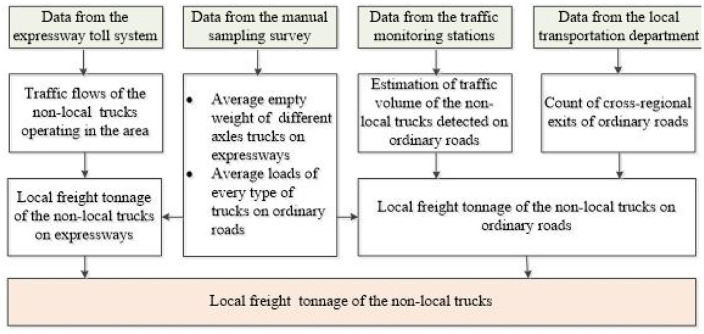
Statistic procedure of the local freight tonnage of non-local trucks.

Statistics of the local freight tonnage of the non-local trucks as shown in Fig 5:

Step 1: Identifying the traffic flows of the non-local trucks operating in the region from the expressway toll system, and extracting key information such as the total weight of vehicle and goods.
Step 2: Removing the empty trucks according to the average empty weight of trucks with different number of axles from the manual sampling survey, and then estimating the local freight tonnage of the non-local trucks on expressways.
Step 3: Estimating the traffic volume of the non-local trucks from the traffic monitoring stations at cross-regional exits of ordinary roads.
Step 4: Estimating the local freight tonnage of the non-local trucks on the ordinary roads where the traffic monitoring stations are deployed based on the average loads of each type of truck on ordinary roads from the manual sampling survey.
Step 5: Estimating all the local freight tonnage of the non-local trucks on ordinary roads via scaling up the local freight tonnage obtained in Step 4 by the count of cross-regional exits of ordinary roads.
Step 6: Calculating the local freight tonnage of the non-local trucks by simple addition.

#### 2.2.2 Statistic of local freight ton-kilometers

**Fig 6:**
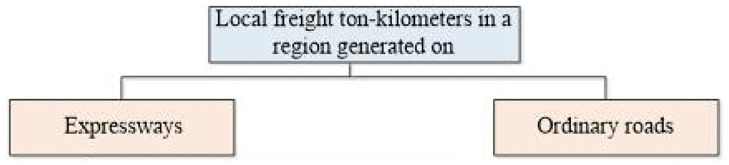
Composition of local freight ton-kilometers.

As shown in Fig 6, we divide the regional road network into two parts: the expressway network and the ordinary road network, and conduct statistics of the local freight ton-kilometers on expressways and ordinary roads respectively.

**Fig 7:**
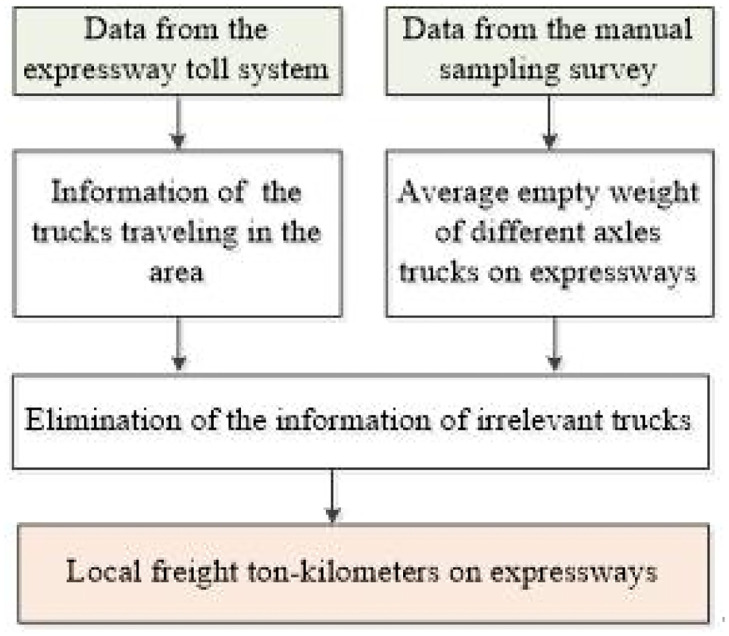
Statistic procedure of the local freight ton-kilometers on expressways.

Statistics of the local freight ton-kilometers on expressways as shown in Fig 7:

Step 1: Extracting key information of the trucks traveling in the region such as the full sample truck flows, total weight of vehicle and goods, and distance traveled from the expressway toll system.
Step 2: According to the average empty weight of trucks with different numbers of axles on expressways from the manual sampling survey, the irrelevant truck flows from the full sample truck flows are removed, which include the empty trucks and the trucks crossing the region without loading and unloading (neither the loading place nor the unloading place of trucks is local).
Step 3: Estimating the local freight ton-kilometers on expressways based on the data extracted above.

**Fig 8:**
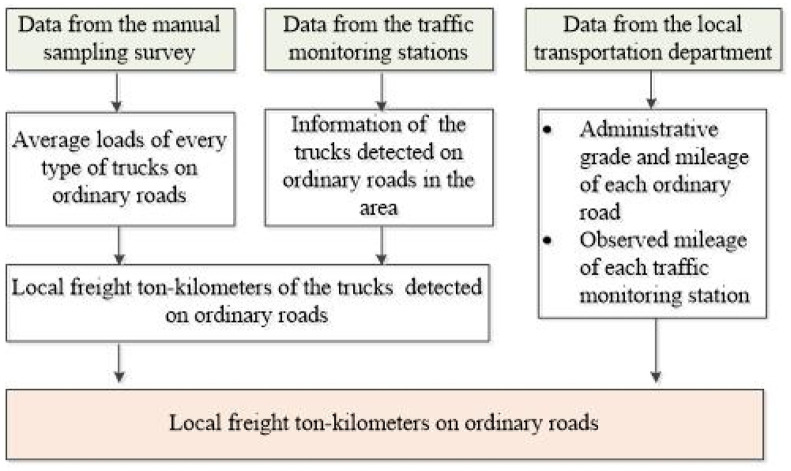
Statistic procedure of the local freight ton-kilometers on ordinary roads.

Statistics of the local freight ton-kilometers on ordinary roads as shown in Fig 8:

Step 1: Extracting key information such as the traffic flows of all types of trucks from the traffic monitoring stations deployed on ordinary roads. In general, there are no trucks crossing the region without loading and unloading on ordinary roads. Thus, it is assumed that all the freight ton-kilometers generated on ordinary roads is local.
Step 2: Extracting the average loads of each type of truck on ordinary roads from the manual sampling survey.
Step 3: Estimating the local freight ton-kilometers on the ordinary roads utilized with traffic monitoring stations based on the data obtained above.
Step 4: Obtaining the administrative grade of each ordinary road utilized with the traffic monitoring station and the observed mileage of each traffic monitoring station from the local transportation department. Also, capturing the mileage of all roads with different administrative grades in the region.
Step 5: Calculating the local freight ton-kilometers on ordinary roads by expanding samples.

## 3 Estimation methods

In this section, several estimation models combined with the statistical process described earlier are developed.

### 3.1 Estimation models of local freight tonnage

As mentioned previously, the local freight tonnage consists of three parts that are not repeated. Let *T*_*c*_ be the local freight tonnage of the local commercial trucks, *T*_*n*_ be the local freight tonnage of the local non-commercial trucks, and *T*_*nc*_ be the local freight tonnage of the non-local trucks. Thus, the local freight tonnage in a region, denoted as *T*, can be got by

$$\begin{array}{c} T=T_{c}+t_{n}+T_{nc}. \end{array}$$ (1)

Surveys and interviews show the local commercial trucks hardly run only on ordinary roads of other regions, which usually pass by expressways. In this case, the freight tonnage generated by the local commercial trucks in other regions is estimated based on the expressway toll system. In addition, all the freight tonnage of the local commercial trucks, denoted as *T*_*yc*_, can be obtained directly from the local statistics yearbook. Thus, the local freight tonnage of the local commercial trucks is got by

$$\begin{array}{c} T_{c}=T_{yc}-\sum_{i=2}^{6}\sum_{k=1}^{n_{ci}}\left(q_{ik}-q_{ei}\right), \end{array}$$ (2)

where *i* is the number of truck axles, *k* is the *k*th trip of local *i*-axle trucks loaded with goods on expressways of other regions, *n*_*ci*_ is the total count of trips of local *i*-axle trucks loaded with goods on expressways of other regions, *q*_*ik*_ is the total weight of vehicle and goods of the *k*th trip, *q*_*ei*_ is the average empty weight of *i*-axle trucks.

In general, the local non-commercial trucks are engaged in short distance transportation, all the trips of the local non-commercial trucks are considered to be an integral part of the local economy, the corresponding freight tonnage generated is considered to be local freight tonnage. Let *N*_*c*_ be the count of the local commercial trucks, and *N* be the total count of the local trucks. Thus, the local freight tonnage of the local non-commercial trucks can be interpreted by

$$\begin{array}{c} T_{n}=\lambda \frac{N-N_{c}}{N_{c}}T_{yc}, \end{array}$$ (3)

where λ is the average load differentiation coefficient between the local commercial trucks and the local non-commercial trucks, which is captured by

$$\begin{array}{c} \lambda =\frac{\sum_{i=2}^{6}\sum_{h=1}^{n_{ih}}\left(q_{ih}-q_{ei}\right)/\sum_{i=2}^{6}n_{ih}}{\sum_{i=2}^{6}\sum_{f=1}^{n_{if}}\left(q_{if}-q_{ei}\right)/\sum_{i=2}^{6}n_{if}}, \end{array}$$ (4)

where *h* is the *h*th trip of the local non-commercial *i*-axle trucks with carrying goods on expressways, *n*_*ih*_ is the total count of trips of the local non-commercial *i*-axle trucks with carrying goods on expressways, *q*_*ih*_ is the total weight of vehicle and goods of the *h*th trip, *f* is the *f*th trip of the local commercial *i*-axle trucks with carrying goods on expressways, *n*_*if*_ is the number of trips of the local commercial *i*-axle trucks with carrying goods on expressways, *q*_*if*_ is the whole weight of vehicle and goods of the *f*th trip.

In order to estimate the local freight tonnage of the non-local trucks more accurately, the local freight tonnage of the non-local trucks are divided into two categories: the local freight tonnage generated by the non-local trucks passing expressways, and the local freight tonnage generated by the non-local trucks without passing expressways. That is, the local freight tonnage generated by the non-local trucks passing through both expressways and ordinary roads is included in the category of the local freight tonnage generated by the non-local trucks passing expressways. The local freight tonnage generated by the non-local trucks passing expressways, denoted as *T*_*nce*_, is represented by

$$\begin{array}{c} T_{nce}=\sum_{i=2}^{6}\sum_{m=1}^{n_{im}}\left(q_{im}-q_{ie}\right), \end{array}$$ (5)

where *m* is the *m*th trip of the non-local *i*-axle trucks on the expressways in the region, *n*_*im*_ is the total count of trips of the non-local *i*-axle trucks on the expressways in the region, *q*_*im*_ is the total weight of vehicle and goods of the *m*th trip.

Surveys and interviews show that the non-local trucks hardly always run on ordinary roads within the region, which are usually engaged in trans-regional transport. In this case, the local freight tonnage generated by the non-local trucks on ordinary roads is estimated based on the truck trips of trans-regional transport. As we know, not all the ordinary roads at the boundary of the area have traffic monitoring stations, all the local freight tonnage generated by the non-local trucks on ordinary roads can be obtained via scaling up the freight tonnage within the observation range of traffic monitoring stations by the number of cross-regional exits of ordinary roads.

In addition, different from the expressway toll system, the traffic monitoring stations only identify the traffic volume of different types of trucks, not the total weight of vehicle and goods. It is crucial to obtain the average loads of each type of truck through the manual sampling survey. Thus, the local freight tonnage generated by the non-local trucks on ordinary roads, denoted as *T*_*nco*_, is interpreted by

$$\begin{array}{c} T_{nco}=\sum_{g=1}^{4}\frac{N_{go}}{M_{go}}\sum_{r=1}^{n_{gr}}\sum_{j=1}^{4}a_{grj}q_{oj}, \end{array}$$ (6)

where *g* is the administrative grade of the ordinary roads set up with traffic monitoring stations, and which includes four types: national highway, provincial highway, county highway and township highway. *N*_*go*_ is the total count of cross-regional exits of ordinary roads with the administrative grade *g*, *M*_*go*_ is the count of cross-regional exits of ordinary roads with the administrative grade *g* where the traffic monitoring stations are deployed, *r* is the *r*th traffic monitoring station at cross-regional exits of ordinary roads with the administrative grade *g*, *n*_*gr*_ is the total count of the traffic monitoring stations at cross-regional exits of ordinary roads with the administrative grade *g*, *j* is the truck type on ordinary roads, *a*_*grj*_ is the traffic volume of *j*-type trucks detected at the *r*th traffic monitoring station deployed on ordinary road with the administrative grade *g*, *q*_*oj*_ is the average loads of *j*-type trucks on ordinary roads.

As a result, the local freight tonnage of the non-local trucks is got by

$$\begin{array}{c} T_{nc}=T_{nce}+T_{nco}. \end{array}$$ (7)

### 3.2 Estimation models of local freight ton-kilometers

As mentioned in the previous section, the local freight ton-kilometers is composed of two parts. Let *TK*_*e*_ be the local freight ton-kilometers on expressways, and *TK*_*o*_ be the local freight ton-kilometers on ordinary roads. Thus, the local freight ton-kilometers in a region, denoted as *TK*, is represented by

$$\begin{array}{c} TK=TK_{e}+TK_{o}. \end{array}$$ (8)

Since the key information of every trip of all trucks on expressways from the expressway toll system, the local freight ton-kilometers on expressways with full sample is estimated, which can be got by

$$\begin{array}{c} TK_{e}=\sum_{i=2}^{6}\sum_{m=1}^{n_{i}}\left(q_{im}-q_{ei}\right)s_{im}, \end{array}$$ (9)

where *m* is the *m*th trip of *i*-axle trucks loaded with goods on expressways, *n*_*i*_ is the total count of trips of *i*-axle trucks loaded with goods on expressways, *q*_*im*_ is the total weight of vehicle and goods of the *m*th trip of *i*-axle trucks loaded with goods, *s*_*im*_ is the distance travelled of the *m*th trip of *i*-axle trucks.

Since the origin-destination matrix of all trucks on ordinary roads is hard to obtain, it is necessary to calculate the local freight ton-kilometers on ordinary roads via scaling up the freight ton-kilometers within the observation range of traffic monitoring stations by mileage. So, the local freight ton-kilometers on ordinary roads is interpreted by

$$\begin{array}{c} TK_{o}=\sum_{g=1}^{4}\frac{N_{g}}{M_{g}}\sum_{d=1}^{n_{g}}\sum_{j=1}^{4}a_{gdj}q_{oj}s_{gd}, \end{array}$$ (10)

where *N*_*g*_ is the total mileage of ordinary roads with the administrative grade *g* in the region, *M*_*g*_ is the mileage of ordinary roads with the administrative grade *g* that can be detected by the traffic monitoring stations, *d* is the *d*th traffic monitoring station, *n*_*g*_ is total count of traffic monitoring stations on ordinary roads with the administrative grade *g*, *a*_*gdj*_ is the count of trips of *j*-type trucks detected at the *d*th traffic monitoring station deployed on ordinary road with the administrative grade *g*, *s*_*gd*_ is the mileage detected by the *d*th traffic monitoring station deployed on ordinary road with the administrative grade *g*. What needs illustration is that *N*_*g*_ can be obtained directly from the local transportation department, and *M*_*g*_ can be calculated by

$$\begin{array}{c} M_{g}=\sum_{d=1}^{n_{g}}s_{gd}. \end{array}$$ (11)

## 4 Case study

In this section, Sichuan province as an example is taken to carry out freight statistics on the local freight tonnage and local freight ton-kilometers. As shown in Table 1. In 2019, there are 7523 kilometers of expressways, 17372 kilometers of ordinary national roads, 21420 kilometers of ordinary provincial roads, 22511 kilometers of ordinary county roads and 49688 kilometers of ordinary township roads in Sichuan province. 1799 traffic monitoring stations are deployed on the entire road network, and the total mileage of ordinary roads detected by the traffic monitoring stations reaches 40400 kilometers, which accounts for 36.4 percent of all ordinary roads of Sichuan province. A manual sampling survey extensively is carried out and 438327 samples are obtained, the average empty weight of trucks with different numbers of axles are shown in Table 2, and the average loads of every type of trucks on ordinary roads are shown in Table 3. In addition, Sichuan Province has a total of 1.153 million trucks in 2019, 822000 of which are commercial trucks.

**Table 1:** Roads mileage of Sichuan province in 2019.

Types of roads	Expressways	National roads	Provincial roads	County roads	Township roads
Kilometers	7523	17372	21420	22511	49688

**Table 2:** Average empty weight of trucks with difirrent numbers of axles on expressways.

Axle-based classification	2-axle trucks	3-axle trucks	4-axle trucks	5-axle trucks	6-axle trucks
Average empty weight (tons)	6.58	11.44	14.36	17.90	19.07

**Table 3:** Average loads of every type of trucks on ordinary roads.

Truck type	small trucks	medium trucks	heavy trucks	very heavy trucks
Average loads (tons)	2.09	6.29	12.43	24.72

Tables 4 and 5 show the local freight tonnage and local freight ton-kilometers of Sichuan province in 2019. Compared with the official freight data from Sichuan Statistics Yearbook, the local freight tonnage is 360 million tons more than the official data, and the local freight ton-kilometers is also 98.28 billion ton-kilometers more than the official data. That is, a huge amount of local freight tonnage and local freight ton-kilometers related to the local economy is not included in the existing freight statistics. In particular, the freight tonnage generated by the local non-commercial trucks reaches 474 million tons, and accounts for 23.9% of all the local freight tonnage. In fact, the more developed the real economy, the more the local non-commercial trucks. The freight tonnage shipped by non-commercial trucks accounts for more than 28% of all freight tonnage in Germany [20]. The freight tonnage generated by the non-commerial trucks engaged in agriculture, forestry, animal husbandry, fishery, construction, wholesale and retail trade in the United States is more than twice that generated by commercial trucks [21]. These imply that it is essential to accomplish statistics of the freight tonnage generated by non-commercial trucks. The more the freight tonnage generated by non-commercial trucks, the greater the deviation between the official road freight data and the real road freight performance at a subnational level.

**Table 4:** Local freight tonnage of Sichuan province in 2019.

Contents	*T*	*T*_*c*_	*T*_*n*_	*T*_*nc*_
Million tons	1987	1508	474	195

**Table 5:** Local freight ton-kilometers of Sichuan province in 2019.

Contents	*TK*	*TK*_*e*_	*TK*_*o*_
Billion ton-kilometers	251.08	94.63	156.45

In addition, we can see from the statistical results that the freight tonnage generated by the local commercial trucks running on expressways of other provinces is less than that generated by the non-local trucks within Sichuan province. That is, the participation of non-local trucks in the freight transport activities in Sichuan is high, which is related to the level of economic development in Sichuan. For instance, the number of trucks of Guizhou province participating in Sichuan freight transport activities is 2.3 times that of Sichuan province participating in Guizhou province, since the economic development level of Sichuan province is higher than that of Guizhou province. This indicates that the higher the level of local economic development, the higher the degree of non-local trucks participating in local freight transport activities. In fact, this phenomenon is common all over the world. For example, as the most developed country in Europe, 65 percent of German imports and exports are carried by the trucks registered in other countries [22].

## 5 Conclusions

In order to more accurately and objectively reflect the regional road freight transportation situation at a subnational level, aiming at the problem of deviation in the current road freight statistics in China, a new concept of the local freight tonnage and ton-kilometers is proposed in this paper, which are closely related to local economic activities. The statistic procedures of local freight tonnage and local freight ton-kilometers are presented in detail, and the estimation models of local freight tonnage and local freight ton-kilometers are also established based on five basic datasets obtained easily. Finally, a case study in Sichuan province of China is conducted.

The statistical results show that both the local freight tonnage and the local freight ton-kilometers of Sichuan province are more than the official freight data from Sichuan Statistics Yearbook, because there is a good deal of local freight tonnage and local freight ton-kilometers generated by the local non-commercial trucks and non-local trucks that are not included in the existing road freight statistics. This reveals that it is very necessary to carry out statistical research on the local freight tonnage and local freight ton-kilometers, so as to provide more accurate and scientific references for the decision-making of various transportation plans, subsidy policies, infrastructure investments and so on. In addition, results shows that the more developed the real economy, the more the local non-commercial trucks. The higher the level of local economic development, the higher the degree of non-local trucks participating in local freight transport activities. These indicate that the higher the level of local economic development, the greater the deviation between the official road freight data and the real road freight performance at a subnational level.

This study is an innovative attempt at road freight statistics based on the territoriality principle, where either the origin or the destination of goods transported is local. And there is still inadequate in some respects. For example, to obtain all freight data for the entire ordinary roads, it is necessary to scale up the freight data from traffic monitoring stations by mileage and number of cross-regional exits. Because the current monitoring range of traffic monitoring stations does not cover all ordinary roads. In the future, it is an important research direction to continue to carry out freight statistics on important transport corridors based on the territoriality principle, which can be used to better guide the construction of infrastructure on transport corridors and the freight route planning.

## Supporting information

S1 Table GOD path assignment query between OD points (part). (XLSX)
